# Acute Appendicitis after Liver Transplantation: A Case Report and Review of the Literature

**Published:** 2017-11-01

**Authors:** S. E. Sheppard, H. L. Marecki, C. M. Psoinos, B. Movahedi, M. J. Furman, A. Bozorgzadeh, P. N. Martins

**Affiliations:** Department of Surgery, Transplant Division, University of Massachusetts Medical School, Worcester, MA, USA

**Keywords:** Liver transplant, Appendicitis, Appendectomy, Laparoscopy, Abdomen, acute

## Abstract

Acute appendicitis is one of the most common etiologies for acute abdomen. However, fewer than 30 cases of acute appendicitis after liver transplantation have so far been reported in the literature. Previous case studies have concluded that acute appendicitis after liver transplantation may present differently than in non-immunosuppressed patients and thus may lead to more complications. Herein, we describe the fourth case of laparoscopic appendectomy in a 40-year-old female presenting with an acute abdomen, 10 years after orthotopic liver transplantation for autoimmune hepatitis. Additionally, we review the literature, and emphasize the importance for laparoscopic, rather than open appendectomy after liver transplantation. Overall, despite the small number of reported cases of appendicitis after orthotopic liver transplantation, we found the incidence and clinical presentation are similar to patients without liver transplantation. The etiologies for appendicitis in patients after liver transplantation may be different than in those not chronically immunosuppressed, with significantly less lymphoid hyperplasia and increased fecalith and cytomegaloviral infections. Preliminary results showed that laparoscopic appendectomy after liver transplantation results in decreased hospital stays and fewer complications.

## INTRODUCTION

Acute appendicitis is one of the most common etiologies for acute abdomen, but fewer than 30 cases of acute appendicitis after liver transplantation have so far been reported in the English literature [1, 2]. Previous studies have concluded that acute appendicitis after liver transplantation may present differently than in non-immunosuppressed patients and thus may result in more complications. In agreement with this, open appendectomy after exploratory laparotomy has been more prevalent than laparoscopic appendectomy in liver transplant recipients [3-7]. Herein, we present the fourth reported case of laparoscopic appendectomy after liver transplantation and review the previously published case reports to examine the incidence and clinical presentation of appendicitis after orthotopic liver transplantation.

## CASE REPORT

A 40-year-old woman with a history of orthotopic liver transplantation for autoimmune hepatitis 10 years before, currently maintained on mycophenolate mofetil and tacrolimus for chronic immunosuppression, presented to the University of Massachusetts Medical Center with signs and symptoms concerning for acute appendicitis. She reported onset of periumbilical abdominal pain about 24 hours earlier, which had migrated to the right lower quadrant at the time of presentation. She also experienced malaise, nausea, emesis, anorexia, and subjective fever and chills. At the time of presentation, her vital signs were stable, except for tachycardia (rate of 114); she had signs of focal peritonitis in the right lower quadrant on examination. Laboratory evaluation was significant for leukocytosis, elevated to 12,5000/mm^3^ (84.5% neutrophils, 9% lymphocytes, 5.7% monocytes, 0.6% eosinophils, 0.2% basophils) and normal liver function tests (LFT). Computed tomography showed an enlarged fluid-filled appendix with peri-appendiceal inflammation. 

**Figure 1:**
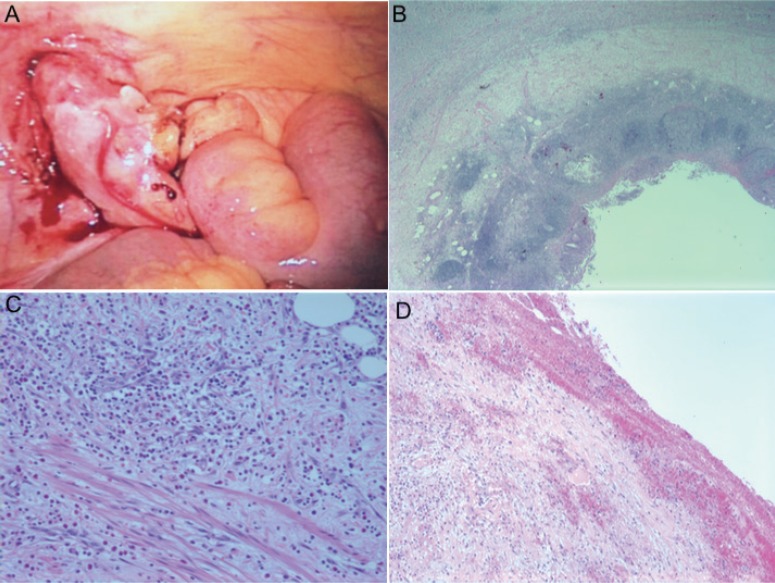
Appendix pathology. A) Gross appendix; B) Low power view of appendix; C) Inflammation extending into mesoappendiceal fat; D) Fibrinoid exudate

The patient was diagnosed with acute appendicitis, started on ceftriaxone and metronidazole. A laparoscopic appendectomy was performed within 12 hours of presentation. Intraoperatively, the appendix appeared inflamed and rigid without any evidence of perforation (Fig 1A). The operation was uneventful. On gross examination, the serosal surface was hemorrhagic with exudate. Microscopy demonstrated typical acute appendicitis and periappendicitis with inflammation extending into the mesoappendiceal fat (Fig 1B-D). Postoperatively, the antibiotics were continued. Mycophenolate mofetil was held during the course of antibiotic therapy. The patient had no complications. LFTs, tacrolimus level, and WBC count were within normal limits and the patient was discharged one day after laparoscopic appendectomy.

## DISCUSSION

The case presented here was the only presentation of acute appendicitis after liver transplant at our center from 1998–2013 in 426 patients undergoing 443 transplantations (17 patients underwent two transplantations), resulting in a rate of 0.23%. Previous case studies showed rates of about 0.09% in the US [5], and 0.49% in China [3]. These statistics are hard to compare as these are cumulative incidences rather than incidence described for one year. In the US, the yearly incidence in non-immunosuppressed patients is about 0.09% [8]; the total lifetime risk is estimated at 0.11% [9]. Despite the slight variation in the rate, these studies suggest that the incidence of appendicitis is similar between in patients with or without liver transplants at less than 0.5%. 

Despite immunosuppression, the majority of liver transplant recipients, including our case, present with classic symptoms of acute appendicitis, such as fever, nausea, emesis, anorexia, periumbilical or right lower quadrant pain, and signs of peritonitis on examination. A retrospective case study of acute appendicitis after liver transplantation concluded that immunosuppression may delay the diagnosis, in part by contributing to the absence of leukocytosis [5]. However, across all cases reported of acute appendicitis after liver transplantation, including the present case, 73% of patients presented with leukocytosis; 27% did not (Table 1) [1-3, 6, 7], similar to the distribution in non-immunosuppressed patients [10].

**Table 1:** Published cases of appendicitis after liver transplantation

Author, year of publication	Abt *et al*, 2005	Savar *et al*, 2005	Ceulemans *et al*, 2010	Wu *et al*, 2011	Aktas, 2011	Quartey, 2012	Wei, 2014	McCarty, 2015	Fonseca—Neto, 2016	Present case
Study size	8	17	1	8	1	1	1	2	5	1
Study period	1996-2004	1997–2004	—	2000—2007	—	—	10 years	—	12 years	1998–2013
Rate of appendicitis	0.09%	0.18%	—	49%	—	—	0.27%	—	54%	23%
Diagnosis:										
Appendicitis	8	15	1	4	1	1	1	2	5	1
Misdiagnosis	—	2	—	4	—	—	0	—	—	0
Age at transplantation	—	1–72 y/o (34)	63 y/o	18–75 y/o (49.5)	2 y/o	27 y/o	52 (below liver only)	42–50 y/o	—	30 y/o
Age at appendectomy	—	7–73 y/o (37)	63 y/o	—	6 y/o	29 y/o	52 d/o	42–50 y/o	15–58 y/o	40 y/o
Interval from Tx to appendectomy	21–5509 days	16–2977 days (1064)	16 days	8–13 days (9.9)	4.5 years	2 yrs	8 mo	6–8 mo	—	10 yrs
Interval from presentation to appendectomy	0.58-3 days	0–4 days (0.94	1 day	1–2 days	—	—	—	<24 hrs	—	<12 hrs
Signs and Symptoms:										
Abdominal pain	87.5%	94%	100%	100%	—	100%	100%	100%	100%	100%
Fever (>38 °C)	75%	—	0%	75	100%	0%	0%	50%	—	0%
Nausea ± emesis	87.5%	88%	—	0%	—	100%	100%	50%	—	100%
Diarrhea	25%	—	—	0%	0%	100%	0%	—	—	100%
RLQ tenderness	—	94%	—	100%	—	100%	100%	100%	40%	100%
Peritoneal signs	87.5%	—	100%	100%	0%	0%	0%	0%	40%	100%
Leukocytosis >10,000/mm^3^	37.5%	76%	100%	100%	100%	100%	0%	0%	—	100
Leukocytosis <10,000/mm^3^	62.5%	24%	0%	0%	0%	0%	100%	0%	—	0%
Diagnostic imaging:										
CT	87.5%	94%	100%	0%	100%	100%	100%	100%	—	100%
US	12.5%	6%	0%	100%	0%	0%	100%	0%	—	0%
Surgical technique										
Open appendectomy (± laparoscopy visualization)	100%	100%	100%	100%	100%	0%	0%	50%	100%	0%
Laparoscopic appendectomy	0%	0%	0%	0%	0%	100%	100%	50%	0%	100%
Pathology										
Inflammation and edema	100%	88%	100%	50%	100%	100%	100%	50%	—	100%
Serositis	0%	6%	0%	0%	—	100%	—	—	—	0%
Normal appendix	0%	12%	0%	50%	0%	0%	—	0%	—	0%
Duration of hospitalization post-op	3-12 days (5.6)	1–20 days (7)	12 days	—	—	1 day	—	6–8 days	2–45 days	1 day
Duration of follow-up	7-74 mo (32.5 mo)	3–2492 days (712 days)	—	1 day–112 mo (49.6 days)	18 mo	—	—	6–12 mo	—	14 days
Complications										
Wound infection	25%	0%	0%	0%	—	0%	0%	0%	0%	0%
Perforation	50%	6%	0%	0%	100%	0%	0%	0%	0%	0%
Intra-abdominal abscess	12.5%	6%	0%	0%	—	0%	0%	0%	40%	0%
Ventral hernia	0%	6%	0%	0%	—	0%	0%	0%	40%	0%
Small bowel obstruction	—	6%	0%	0%	—	0%	0%	0%	0%	0%
Hematuria	—	6%	0%	0%	—	0%	0%	0%	0%	0%
Mortality	0%	0%	0%	0%	0%	0%	0%	0%	0%	0%
Acute rejection during hospitalization	0%	0%	0%	25% (mis dx)	0%	0%	0%	0%	0%	0%

*included 8 liver, 5 heart, 3 kidney, 2 kidney-pancreas, and 1 heart-kidney transplants^rate for 1 liver and 1 kidney Tx. Other statistics for post-liver Tx onlyRanges listed; means in parentheses, Wu *et al*—F/U, signs and symptoms for appendicitis patients only

Imaging may help to confirm the clinical diagnosis of appendicitis. In the present case, CT helped to confirm the diagnosis. Indeed, CT is more sensitive (91% vs 78%) and specific (90% vs 83%) than ultrasound to aid in the diagnosis of acute appendicitis [11]. The published cases of appendicitis after liver transplantation support this meta-analysis, as the group that used ultrasound as the primary imaging modality also had the highest misdiagnosis rate [3].

A case study by Abt, *et al*, suggests perforation may be more frequent, as it was seen in 50% of patients [5]. However, in that report, three of the four patients initially presented with a three-day history of abdominal pain. Therefore, the delay in seeking medical care was likely the cause of perforation, and perhaps unrelated to transplantation status. Evidence of perforation was reported in two single other case reports [7]. However, out of the studies evaluated for this paper, two did not account for perforation [3, 4]. Therefore, because of the paucity of reported cases, it is hard to accurately assess this outcome.

The etiologies for appendicitis in patients after liver transplantation may be different than in those not chronically immunosuppressed. Appendicitis may occur when obstruction of the lumen of the appendix results in increased intraluminal pressure, localized inflammation, and bacterial overgrowth. In younger patients, a viral illness followed by lymphoid hyperplasia is thought to be the main cause of acute appendicitis. In one case study of eight patients with appendicitis after liver transplantation, none of the patients had lymphoid hyperplasia on pathological examination, likely because immunosuppression may lead to decreased lymphoid hyperplasia [5]. In liver transplant recipients, the mean age at the presentation of acute appendicitis is 2 (Table 1) [1, 3, 4, 6, 7], and lymphoid tissue atrophies with age [12], thus providing additional support for an alternative cause. Therefore, acute appendicitis after liver transplantation is likely caused by particulate matter including a fecalith obstructing the lumen. Cytomegalovirus (CMV) infection may also be a cause of acute appendicitis after solid organ transplantation, such as kidney [13, 14], but it has only been reported once in a liver transplant recipient [15]. A biopsy with microscopic assessment is required to establish a definitive diagnosis of tissue invasive CMV, so it is important to consider CMV as a potential cause of appendicitis in a transplant recipient and test accordingly to ensure effective postsurgical treatment with antivirals. Future case studies of appendicitis after liver transplantation in pediatric patients may yield further insights into immunosuppression inhibiting lymphoid hyperplasia, as this is the main mechanism in younger patients (<20 years of age), which contribute to almost one-third of all cases of appendicitis [8]. 

Although laparoscopic appendectomy has been shown to be beneficial compared to open appendectomy in the treatment of appendicitis there are surprisingly few reported cases. Laparoscopic appendectomy results in decreased post-operative pain, shorter post-operative hospital stays, decreased wound infection, and faster return to normal activity and diet [15]. In addition, it may help to diagnose and treat other causes of acute abdomen. Given that appendicitis after transplantation will likely be a complex presentation, laparoscopy is likely both cost-effective and helps minimize complications [16]. Despite this, surprisingly, the current report is only the third published case of laparoscopic appendectomy after liver transplantation. This may be related to the fear of perforation in transplant recipients. One author suggested exploratory laparotomy may be better if the diagnosis of appendicitis is uncertain, given the potential for other pathologies such as intestinal perforation or biliary leakage to present similarly [3]. Interestingly, the limited data show on average decreased hospital stays and fewer complications in patients who have undergone laparoscopic appendectomy after liver transplantation (**Table 1**). 

In conclusion, we found that acute appendicitis after liver transplantation has similar presentation, incidence rate, and outcome compared to non-transplant patients and laparoscopic appendectomy should be considered when appropriate. 
